# Integrating nutrition and epigenetics in precision medicine for Alzheimer's disease

**DOI:** 10.1002/alz70860_100840

**Published:** 2025-12-23

**Authors:** Shampa Ghosh, Krishna Kumar Singh, Manchala Raghunath, Jitendra Kumar Sinha

**Affiliations:** ^1^ GloNeuro Academy, Noida, Uttar Pradesh, India; ^2^ Symbiosis International (Deemed University), Pune, Maharastra, India; ^3^ ICMR ‐ National Institute of Nutrition, Hyderabad, Telangana, India

## Abstract

**Background:**

Alzheimer's disease (AD) is a progressive neurodegenerative disorder influenced by a complex interplay of genetic, environmental, and lifestyle factors. Traditional approaches targeting AD's late stages have shown limited success in altering its progression. Precision medicine, which integrates genetics, epigenetics, and lifestyle modifications, including nutrition, represents a promising paradigm for early diagnosis, personalized risk stratification, and tailored interventions. Nutrition has emerged as a critical modifiable factor in AD, with dietary components influencing epigenetic mechanisms that regulate gene‐environment interactions contributing to disease onset and progression.

**Method:**

This article synthesizes current evidence on the role of nutrition and epigenetics in AD management, focusing on dietary interventions and their impact on epigenetic mechanisms such as DNA methylation, histone modification, and non‐coding RNA regulation. The integration of nutritional data with biomarker innovations, including plasma phosphorylated tau and neurofilament light chain, alongside genetic profiling tools like APOE‐ε4 genotyping, is explored. Systems biology and machine learning approaches are highlighted for their role in combining multi‐omic and nutritional data to develop predictive and personalized therapeutic strategies.

**Result:**

Nutritional components such as omega‐3 fatty acids, polyphenols, vitamins, and antioxidants were found to influence key epigenetic pathways, reducing neuroinflammation, oxidative stress, and synaptic dysfunction associated with AD. Biomarker advancements have enabled earlier detection of AD pathology, while genetic insights offer refined risk stratification. Integrating nutrition into these frameworks enhances precision medicine by addressing modifiable risk factors and tailoring interventions to individual patient profiles. Emerging systems biology approaches show significant potential for generating personalized care strategies that incorporate dietary modifications as a central component.

**Conclusion:**

Integrating nutrition and epigenetics into precision medicine offers a transformative pathway for addressing Alzheimer's disease's complexity. Dietary interventions, by modulating epigenetic processes, provide a feasible and impactful strategy for reducing AD risk and progression. Combining nutritional insights with genetic and biomarker data through advanced analytical tools can improve early detection, optimize treatment efficacy, and enhance patient outcomes. Despite challenges such as cost and accessibility, this integrative approach underscores the importance of holistic, patient‐centric strategies in combating AD and other neurodegenerative diseases.

